# Recent Advances in Orthopædics

**Published:** 1913-10-18

**Authors:** 


### RECENT ADVANCES IN ORTHOPÆDICS.

### Some Modern Methods and their Application.

The Orthopædic Section of the International Medical Congress discussed many interesting points, and the bare enumeration of the subjects shows how wide is the field in this specialty which is only slowly "coming into its own" in England. The work of the section should stimulate attention in orthopædics in this country. Already some very excellent work, both of a practical nature and of what may be ranked as theoretical, has been done here; but the specialty needs more undivided attention. Those interested in it would do well to consider the advisability of starting a special Orthopædic Section in connection with the Royal Society of Medicine. Some years ago there was talk of establishing such a section, but, owing to the want of interest exhibited, the proposals fell through. In the belief that British surgeons are now realising the extent to which foreign surgery has got ahead of them in this subject, we intend to publish a short series of articles indicating recent progress and the most promising lines of future advance.

### Sciatic Scoliosis and Spastic Paraplegia.

Some years ago Goldthwaite drew attention to a form of scoliosis which is associated with sciatic pain, and which was dependent, apparently, on pelvic deformity. The subject has since attracted considerable attention, especially among French orthopædists. Their views were explained at the sectional meeting by Denuce, of Bordeaux, who suggested that the sciatic pain was secondary, and dependent on the scoliosis. The Germans and Americans support the view that the condition is primarily one of spinal osteoarthritis, which leads finally to pelvic distortion, and so produces the sciatica. The condition is in need of further elucidation, since at present the treatment is largely empirical owing to the want of thorough knowledge with regard to its pathology.

Spastic paraplegia engaged the attention of three reporters—Professor Küttner, of Breslau; Professor Vulpius, of Heidelberg; and Mr. Muirhead Little, of the Royal National Orthopædic Hospital. The condition is one which has attracted the attention of orthopædists in all quarters of the world, and much interesting work has been done in regard to its pathology and treatment. The progress made in treatment has, indeed, been astonishing. Ten years ago a case of spastic paraplegia was regarded as almost hopeless from a surgical point of view. All that the surgeon could do was to restore some measure of shape to the deformed limb; the spasticity and the tremors, where they existed, were regarded as incurable. Since then a great deal has been done to make the condition of sufferers more bearable. Nerve transplantation and implantation have been successful in restoring some measure of function to the paralysed limbs; the pioneering efforts of Hoffa to overcome the spasticity and remove the disagreeable tremors have been followed up by his school and by American and Italian workers. The discussion at the sectional meeting ranged round the utility of these modern methods of treatment. It was generally agreed that much good could be achieved by conservative methods, aided by judicious tenotomies and tendon transplantation, while the more modern methods, such as nerve alterations, were more suitable for properly selected cases. Unfortunately the chief exponents of the modern school were not represented at the meeting, neither Professor Lange nor Professor Spitzy taking part in the discussion. A point on which great stress was laid was this, that, no matter what method is chosen in any particular case, the greatest care and patience are necessary in the after treatment, especially in regard to educating the muscles. Without such educative treatment, instruments and surgical measures are merely palliative, and inevitably fail in the long run.

### Scoliosis and Hip Deformities.

Considerable and interesting discussion ranged round that much debated subject, the treatment of scoliosis. Some years ago the Klapp method was regarded as one of the best methods of treatment in certain cases; it has never, however, obtained much popularity in this country, perhaps because the technique of the treatment was not perfectly understood. It has become clear that some rearrangement in the classification of scoliosis is necessary. Dr. Lovett, who is perhaps the greatest transatlantic authority on the subject, sharply differentiates between true or structural scoliosis and postural or muscular scoliosis, a distinction which has been well recognised. Where, however, he differs from some authorities is in his denial of the utility of physical exercises to cure the former variety. Muscular scoliosis is essentially a "habit disease," induced largely by the assumption of faulty carriage of the trunk owing to fatigue. The treatment of such a condition aims both at restoring the normal attitude of the trunk and at safeguarding the muscles of the spine from further fatigue. Here physical exercises, carefully carried out, are of undoubted utility. But some of us have always considered that the structural variety is also amenable to judicious treatment by physical exercise, and that there is indeed no sharp line of demarcation possible between bad conditions of the muscular variety and slight degrees of the more serious structural type. Considerable interest centres round the Abbott method of treatment, which is now very generally employed, and which Dr. Abbott explained and demonstrated at the meeting. The principle of this new and highly original method is over-correction of the deformity while the spine is flexed, and maintaining this over-correction by the use of plaster jackets until the structures have regained their normal position. The new method has been very successful in grave cases which have not yielded to other methods.

Mr. Jackson Clarke opened the discussion on the treatment of congenital dislocation of the hip by the open method. Since the introduction of the usually (in skilled hands) very successful Lorenz method of reposition, the open method has been losing its popularity owing to the indifferent results obtained by even the most expert operators. Mr. Clarke exposes the capsule behind, roughens the acetabulum rim, and shortens the relaxed upper part of the capsule by suturing; in this way he has obtained success in cases which have not been amenable to the "closed method."

No congress seems complete without a discussion of tuberculous joints, and it was exhaustively considered at the sectional meeting. The main original contribution was made by Dr. Albee, of New York, who described and demonstrated his operation for the treatment of spinal caries. The lay press devoted considerable attention to this operation, which, indeed, may claim to be original and striking in the extreme, and to have been singularly successful in Dr. Albee's able hands. Briefly described, it consists in inserting a bony wedge strip, taken from the patient's tibia, into a cleft prepared for its reception in the affected vertebræ by splitting their spinous processes. With proper aseptic precautions this secures a rigid fixation which succeeds in giving that required rest and immobility so indispensable for cure. Albee has performed this operation 145 times, and his success has certainly been most striking, so that this new method may be taken as having definitely established its position as a justifiable procedure in suitable cases.

